# A Hybrid Approach for Alluring Ads Phishing Attack Detection Using Machine Learning

**DOI:** 10.3390/s23198070

**Published:** 2023-09-25

**Authors:** Muhammad Waqas Shaukat, Rashid Amin, Muhana Magboul Ali Muslam, Asma Hassan Alshehri, Jiang Xie

**Affiliations:** 1 Department of Computer Science, University of Engineering and Technology, Taxila 47050, Pakistan; 2 Department of Computer Science, University of Chakwal, Chakwal 48800, Pakistan; 3 Department of Information Technology, College of Computer and Information Sciences, Imam Mohammad Ibn Saud Islamic University, Riyadh 11432, Saudi Arabia; mmmuslam@imamu.edu.sa; 4 Durma College of Science and Humanities, Shaqra University, Shaqra 11961, Saudi Arabia; 5 Department of Electrical and Computer Engineering, The University of North Carolina at Charlotte, 9201 University City Blvd, Charlotte, NC 28223, USA

**Keywords:** website phishing detection, machine learning, alluring ads phishing, URL features, website text analysis, NLP

## Abstract

Phishing attacks are evolving with more sophisticated techniques, posing significant threats. Considering the potential of machine-learning-based approaches, our research presents a similar modern approach for web phishing detection by applying powerful machine learning algorithms. An efficient layered classification model is proposed to detect websites based on their URL structure, text, and image features. Previously, similar studies have used machine learning techniques for URL features with a limited dataset. In our research, we have used a large dataset of 20,000 website URLs, and 22 salient features from each URL are extracted to prepare a comprehensive dataset. Along with this, another dataset containing website text is also prepared for NLP-based text evaluation. It is seen that many phishing websites contain text as images, and to handle this, the text from images is extracted to classify it as spam or legitimate. The experimental evaluation demonstrated efficient and accurate phishing detection. Our layered classification model uses support vector machine (SVM), XGBoost, random forest, multilayer perceptron, linear regression, decision tree, naïve Bayes, and SVC algorithms. The performance evaluation revealed that the XGBoost algorithm outperformed other applied models with maximum accuracy and precision of 94% in the training phase and 91% in the testing phase. Multilayer perceptron also worked well with an accuracy of 91% in the testing phase. The accuracy results for random forest and decision tree were 91% and 90%, respectively. Logistic regression and SVM algorithms were used in the text-based classification, and the accuracy was found to be 87% and 88%, respectively. With these precision values, the models classified phishing and legitimate websites very well, based on URL, text, and image features. This research contributes to early detection of sophisticated phishing attacks, enhancing internet user security.

## 1. Introduction

In this modern era, the advancements of the internet provide immense opportunities for businesses and individuals, which have become a vital part of life. With all this influence, opportunities, and growing internet usage, many online threats are also emerging [1]. The transformation of digital technology is inventing new ways to make the internet secure, but at the same time, the ways of online attacks are also becoming more sophisticated. Among these modern types of online attacks, phishing is one of the most prevalent, in which sensitive information is stolen, which causes financial as well as reputational damage. The phishers try to deceive the end user by impersonating them. In this type of attack, a mimicked page of a legitimate website is created, and the victim is lured to put their sensitive information in that legitimate look-alike attacker’s website. When the victim uses that deceiving website, considering it a genuine one, the attack is successful, and the attacker steals the information provided by the victim. This attack is usually executed through emails, ads, URLs, and malicious software. Spear phishing, email phishing, malware phishing, website phishing, and ads phishing are common phishing attacks [2]. Phishing attacks are common due to the very resemblance of a deceiving website to a legitimate website and the users being unaware of it. It is seen that these attacks are most common for social platforms, online payment websites, and e-commerce websites.

**Figure 1:**
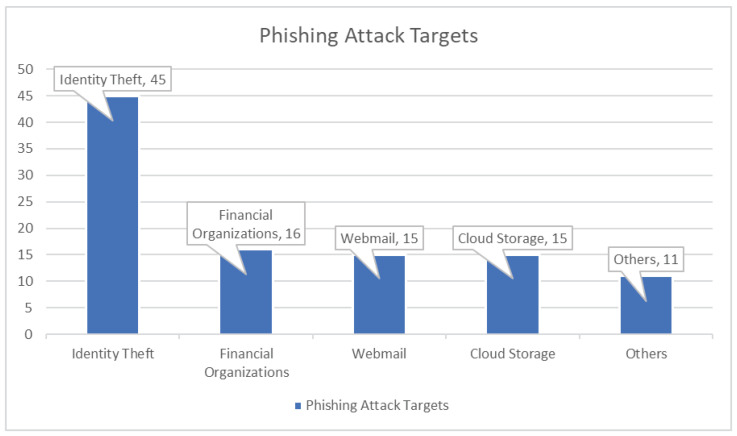
The graph shows targets of phishing attacks.

As shown in Figure 1, according to the Anti-Phishing Working Group, the payment system is the most common target for identity theft, at 45%, followed by financial institutions at 16%, web-mail at 15%, and cloud storage at 15% of the total [3]. In a phishing attack, the user is deceived by taking advantage of the visual resemblance between the fake phishing website created for the attack and the genuine website the victim intended to use. A website that looks identical to some genuine website is created, and the link of such a fraudulent website is sent to the victim in multiple ways [4]. It is seen that victims are lured to click the link by showing them different offers, like discounts, free coupons, lucky draws, and opportunities to increase the number of followers on their social platforms [5]. It is also seen that such phishing links come with content that demonstrates a sense of urgency and fear—telling the victim that their account is being compromised or logged out and to secure an account or logging in back to the account by clicking the link is a usual example. Most of the phishing attacks are executed through emails. In email phishing, the links are sent to the victim, and the phishing process initiates upon clicking the links. These phishing links can be spread using SMSs, also known as SMS phishing or smishing attacks [6]. The same process performed through telephone lines is known as vishing attacks [7]. In spear phishing attacks, the attackers usually have some information about the victim already, and they use this information to build trust by impersonating some genuine authority while they are fraudsters. In whale phishing attacks, the phishers try to present themselves as seniors and tell the victim to follow instructions, leading to a successful attack. Fake links, tweets, posts, and social media platforms are used in another attack vector known as angler phishing [8]. At the enterprise level, phishing attacks can be more severe, as even one of the organization’s employees can put the security of the whole organization at stake by becoming the victim of such attacks [9].

Phishing attacks aim to deceive victims into trusting malicious websites and exposing sensitive information. Attackers use various tactics, such as offering discounts, fake lotteries, or using urgency to compromise credentials. These tricks often manifest as text-written images in pop-up notifications or ads, leading to phishing sites. To combat such sophisticated phishing attempts, a modern solution is required. It should assess websites based not only on URL features but also on the content, including text and images. Mitigating phishing attacks is critical for internet user security. Traditional approaches use predefined rules based on features like URL structure, SSL certificates, and domain-based features to filter phishing sites. However, attackers have advanced, creating phishing sites that exactly mimic legitimate ones, resulting in the traditional methods being less effective. Reporting of phishing attacks by victims helps, but it is limited to reported URLs, which frequently change. Modern phishing sites constantly update content and layout, easily bypassing predefined rules. As a result, traditional techniques struggle to detect ever-evolving phishing attacks. Advanced solutions are needed to detect and avoid these sophisticated threats.

For addressing these issues effectively, a solution that can adapt to the evolving nature of phishing websites by learning from data is essential, instead of relying on fixed rules. machine-learning-based approaches offer such adaptability. These algorithms learn from extensive data and adjust to changes in phishing websites, making them highly capable of combating modern attacks by efficiently identifying anomalies and patterns. Recent research has employed machine learning for phishing detection by analyzing website URL features. Features like the number of commas, hyphens, dashes, characters, spaces, signs, and subdomains were used to train machine learning models to distinguish phishing sites from legitimate ones. To tackle websites with seemingly normal URL features, some studies incorporated text, layout, and image analysis as additional features for enhanced anomaly detection, providing an additional layer of threat identification. Furthermore, optimizing machine learning models by selecting only essential URL features can improve classification efficiency and robustness. Additionally, there is a research gap in terms of limited training dataset sizes. Exploring machine learning algorithms’ performance metrics on larger, optimized datasets is essential for advancing the field of phishing detection. Also, the approaches that used URL only or text only for evaluation caused the modern attacks to bypass the detection systems.

We have proposed a three-layered approach to detect phishing websites using machine learning. The proposed approach is multi-perspective layered evaluation. The first layer analyzes URL features to detect phishing URLs. The second layer uses natural language processing to examine website text for spam content. The third layer processes images and text from ads to classify website content. We have also expanded the dataset size for improved algorithm performance. This layered evaluation enhances detection efficiency, labeling a website as phishing if any layer flags it. This approach includes models that solely assess URLs, and modern text classification via natural language processing enhances accuracy.

## 2. Related Work

Rao et al. [10] proposed an anti-phishing architecture using machine learning approaches. The selected features were categorized into URL obfuscation features, third-party features, and hyperlink-based features. They used a dataset of 2119 phishing sites from Phish Tank and 1407 legitimate sites from the Alexa Database. The algorithms used were RF, J48, LR, BN, MLP, SMO, AdaBoostM1, and SVM to calculate the metrics of sensitivity, specificity, precision, accuracy, and ERR. According to the results, RF showed a maximum accuracy of up to 0.9931 among all the other classifiers. The dataset was limited to textual objects with no captcha objects, while the attackers can use embedded objects instead of textual objects to bypass anti-phishing attacks.

Suryan et al. [11] proposed a machine learning model that involved almost thirty features based on URL for the classification between phishing and legitimate websites. The dataset of the URLs was obtained from the machine learning repository provided by UCI. The dataset was then dimensionally reduced. They applied the machine learning algorithms of random forest, support vector machine, generalized linear model, generalized additive model, recursive partitioning, and regression trees on the reduced dataset. The RF algorithm with 300 trees provided an accuracy of 96.65 percent and a precision of up to 97.4 percent, being the best algorithm for the scenario. The results can be improved by applying higher-order dimensionality reduction techniques like the variance inflation factor (VIF).

Al-Sarem et al. [12] presented an optimized stacking ensemble method for detecting phishing attacks based on URL features. Different machine learning classifiers, AdaBoost, XGBoost, bagging, GradientBoost, and LightGBM were used, and the generic algorithm was used as the optimizer algorithm in order to apply optimization to the mentioned ensemble machine learning methods. Three datasets of phishing data were obtained from Mendeley and UCI library for machine learning. The optimized ensemble method improved the results, and the accuracy was found to be up to 98.58%. The datasets used for the research were limited, and the results can be more efficient using more appropriate datasets.

Butnaru et al. [13] presented an SVM model of machine learning to detect and block phishing attacks with the help of only a novel combination of URL features. Naïve Bayes, decision tree, random forest, support vector machine, and multilayer perceptron were the algorithms used on the imbalanced dataset obtained from Kaggle and PhishTank. The optimized RF algorithm provided the best results with an accuracy of 99.29 percent and detected phishing attacks well as compared to the Google safe browsing (GSB). The features can be optimized and formulated as novel to enhance the effectiveness of the results.

Cuzzocrea et al. [14] also came up with a website phishing detection technique using a machine learning model. They used the decision tree (DT) algorithm to check the legitimacy of the website. The dataset was obtained from the UCI library and different features of the website were selected, including URL as well as others like IP, Web Traffic, Domain Age, etc. Decision stump, logistic model tree (LMT), RepTree, random forest, Hoeffding tree and J48 algorithms were applied to the dataset. The results showed that J48 and RepTree performed better than the other algorithms. The proposed model was aimed at classifying the website as phishing or legitimate and the model performed very well. Still, the research can be pursued to make the results more effective by further focusing on feature extraction and keeping the infected code in view.

Cristian de Souza et al. [15] proposed a tool that they named “PhishKiller,” proficient in identifying and mitigating phishing attacks through an approach of the proxy, engaged to catch user-accessed addresses and featureless machine learning techniques for classifying the URLs as legitimate or phisher. The URL datasets were obtained from UCI and PhishTank and the data was cleaned. The LSTM algorithm of machine learning was used for the testing and training accompanied by the neural networks in order to avoid further predictions as any infected URL was being added to the maintained database. The results showed up to 98.30% accuracy in the detection of legitimate or phishing websites, and the process took only 81.86 ms to identify and mitigate the malicious website. The featureless approach requires a bulk amount of data for efficient results; hence, there needs to be a well-maintained database for improving the efficiency, and this is a limitation of this featureless approach.

Tharani et al. [16] conducted a study to know how the original URLs are mimicked by the phishers, and for this, they used information gain and chi-squared methods of machine learning for feature selection on a phishing dataset. The aim of their research was to identify the features of URLs that are used by phishers to make them look like the actual URLs. They obtained the phishing dataset from the Mendeley repository and extracted 48 features. The total legitimate URLs were 5000, obtained from Alexa and Common Crawl. In the same way, the same amount of phishing URLs were obtained from PhishTank and OpenPhish. The KNN and SVC algorithms were used for classifying the feature detection of URLs that cause phishing attacks. KNN performed well for up to 10 features, while KNN and SVC performed best when the features were more than 10. The results show that null self-redirect hyperlinks in URL and domain name mismatch are the most common techniques that are used to trick humans into phishing attacks. This research is only limited to identifying the most common techniques that are used by phishing attackers.

Sameen et al. [17] came up with a PhishHaven model that is an AI-generated phishing URL detection system working on the basis of ensemble machine learning as well as lexical feature analysis. HTML URL encoding as lexical features, as well as the URL hit approach for the detection of tiny URLs, was also presented. The ensemble machine learning model resulted in real-time detection of phishing attacks. The final evaluation was performed by the unbiased voting method. The dataset of phishing URLs was obtained through the PhishDeep and PhishTank, while the normal URLs were obtained from Alexa. AdaBoost, bagging, decision tree, extra tree, GradientBoost, KNN, LR, NN, RF, and SVM classifiers were applied, and SVM performed best in terms of 97.68% precision. The overall PhishHaven model was up to 98% accurate. The limitation of this model is that it can only detect URLs based on lexical features and patterns similar to the DeepPhish.

Alsariera et al. [18] applied an AI-based meta-learners classifier consisting of four algorithms of AdaBoost/extra tree, bagging/extra tree, rotation forest/extra tree, and Logit Boost/extra tree. These classifiers are designed using extra-based classifiers. The model was then implemented on the phishing website data with the newest features. The application of meta-learners resulted in the detection accuracy of up to 97% specifically, Logit Boost/extra tree gave the maximum of 97.5758% accuracy. The dataset was obtained from the UCI repository. Efficient feature selection and extraction algorithms can be applied for increased efficiency.

Naaz et al. [19] used IOT datasets for the detection of phishing attacks. The common machine learning algorithms of SVM, RF, DT, NN, and linear models were used to identify the data between legitimate and phishing. The result of these machine learning models was then compared with the previous dataset as well as on different datasets. The obtained dataset was UNSW-NB15, designed by the Australian Center of Cyber Security at UNSW Canberra at their Cyber Range Lab, having the precise conventional traffic that may have been attacked by botnets. The random forest algorithm provided the best accuracy of 96.85%. The features used were having numeric values only, which means that non-numeric values will affect results and efficiency. Hybrid machine learning algorithms are proposed for better efficiency of results.

Sirinavasa et al. [20] stated that cybercriminals seek to obtain access to a victim’s personal information, such as passwords, credit card details, and other similar data by using fake websites, emails, or any other authentic online service. Although there are a lot of methods to recognize phishing websites, such as methods based on third parties, methods based on source code, and methods based on URLs, individuals still fall for fraud. According to the findings of this study, it is possible to identify phishing websites by using word embeddings that make use of plain text and domain-specific terminology that is obtained from the source code. To evaluate the model’s word embeddings, ensemble and multimodal methodologies were used. In trials, they found that using multimodal analysis with domain-specific text achieved a significant TPR of 99.59%, FPR of 0.93%, and MCC of 98.68%, which contributed to an overall accuracy of 99.34%.

Almalaq et al. [21] explored the detection of cyber-attacks in smart power systems to address the critical security challenge. The smart grid systems must be significantly secure as the efficient energy supply depends upon these systems. The vulnerabilities in such systems can cause huge economic and security risks, whether human or natural events induce these. To mitigate such security risks, the authors provide an attack detection model that uses Phasor measurement unit (PMU) data. The features were extracted from the PMU data for assessing anomalies using machine learning and deep learning algorithms. The basic classifier of the random forest was used within the AdaBoost ensemble. The approach was validated by evaluating multiple distinct grid systems’ event case studies. The applied models provide better accuracy and outperform the existing methods with an accuracy and detection rate of 93.6% and an accuracy rate of 93.91%. The exploration of deep learning architecture of applied models and enhancements in optimization of the dataset used can potentially lead to more robust and efficient application of the models. According to the study, there are opportunities to leverage the machine learning model strengths for classifying and detecting anomalies in similar real-world problems.

Chen et al. [22] proposed a solution to mitigate the security risks and vulnerabilities of smart grid systems. With the emergence of automation and sophisticated technological management of energy supply systems, the requirements of security protocols and risk mitigation are important. The authors used network vulnerability assessment criteria and identified vulnerability points originating from cyber-attacks and physical faults. The proposed solution includes the concept of microgrid systems that can be considered protective measures from different cyber-attacks when installed at vulnerability points in a smart grid system. Secondly, the study suggested a multi-layer security protocol. The first layer is based on blockchain technology to ensure fundamental security, while the second layer uses reinforcement learning for real-time data monitoring for enhanced protection. Detecting uncertainties, deploying microgrids at vulnerable points, and multi-layered security remarkably enhance smart grid systems’ security.

The results of similar studies show that machine-learning-based approaches can mitigate cyber-security risks and threats as they can detect anomalies and uncertainties. Also, the insights from similar studies confirm that the previous data can be used to identify features and patterns that can be used to train machine learning algorithms. Due to their learning capabilities, these trained machine-learning models can efficiently detect and mitigate modern security threats and cyber-attacks. The machine learning approaches also can handle the ever-changing modern threats.

## 3. Proposed Solution

There has been research for finding efficient solutions to detect and mitigate phishing websites, but with the evolution of phishing attacks, the approaches have struggled to flag the websites correctly. Our layer-based solution presents the multi-perspective evaluation of phishing websites to minimize the risks of bypassing the phishing detection filters. The proposed layered model evaluates the alluring ads and their origin websites by performing classification based on URL, text, and image content. Our proposed solution comprises of following layered model.

### 3.1. Layered Model

Efficient detection of attacks requires using sufficient features to broaden the scope of website evaluation. For this, we have proposed three layers of website evaluation.

- **Improved URL Layer**: The machine learning approaches based on URL features required optimization as modern phishing website URL features kept updating to develop an identical URL structure to legitimate websites to bypass the anti-phishing filters. To handle this problem, we have proposed more optimized and efficient URL features in our first layer for enhanced evaluation of website URLs to assess it regarding phishing. The detailed architecture of this layer is provided in Section 3.4.

- **Text Layer**: It is seen that the URLs of phishing websites can still be undetectable by phishing filters due to exactly identical structures to the legitimate website URLs. To handle this problem, we have added an additional second layer of text-based classification. This layer evaluates the website text data for detecting any text-based features that belong to the phishing class. The comprehensive details are provided in Section 3.5.

- **Image Layer**: The approaches that used the text content of websites only for evaluating a website struggled to detect spam websites as such websites contain only image content and no text content. To handle this, we propose another layer of evaluation that scrutinizes a website based on image data. The complete architecture of the image layer is provided in Section 3.6.

**Figure 2:**
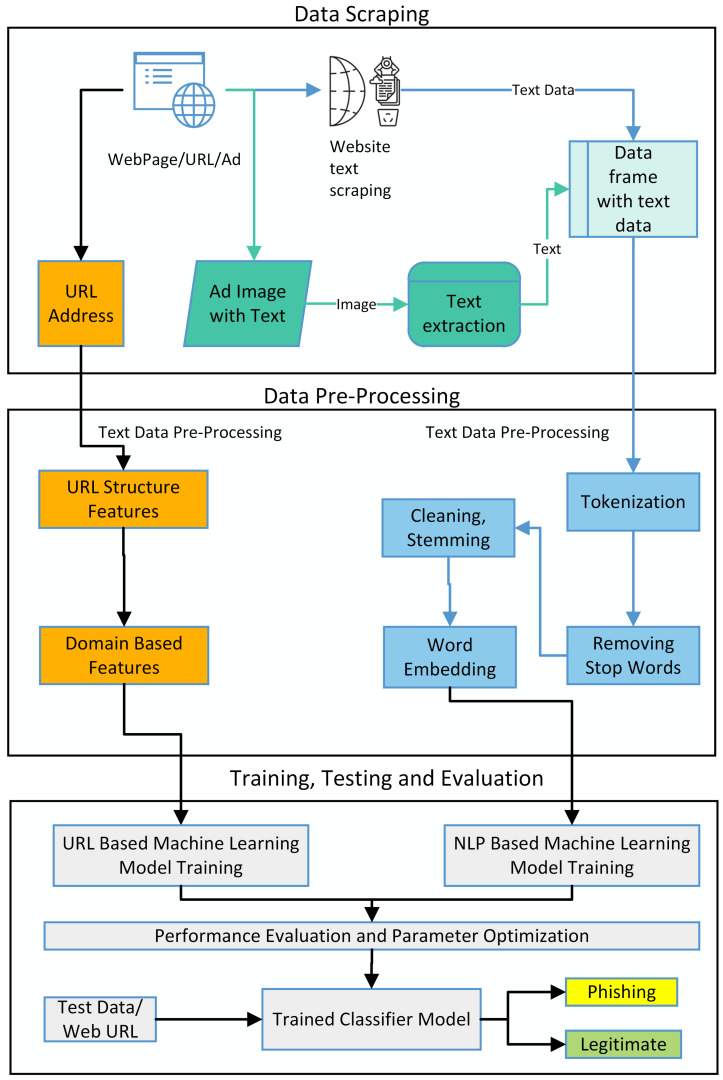
The image shows layered approach of proposed model.

The proposed layered model for the detection of phishing websites is described in Figure 2 below:

The proposed layered model for detecting phishing websites is described in Figure 2. The first layer evaluates the URL of the website. In this layer, the salient features from the URL string are extracted. These extracted features, along with the class labels, are used to train the model. Once the model is trained on this labeled data, the classifier is ready to test a URL based on the salient features upon which the model is trained. The classification model evaluates the features of the provided URL and decides whether it is either a legitimate URL or a phishing URL. In the second layer, the text data from a website is scraped and pre-processed before labeling. The NLP-based machine learning classifier model provides the labeled text data against a URL. The model evaluates the provided text data’s structure, presence, precedence, and patterns. The NLP model learns text data patterns based on this text evaluation and label. These text data patterns are evaluated in the testing phase to classify a website as legitimate or phishing. Similarly, the images are obtained for the websites containing images, and text data are extracted from these images. These extracted text data are pre-processed, and NLP models are trained on this text data, as mentioned in the second layer of text evaluation.

### 3.2. Use Case Scenario

An example describes the use case scenario. Through a search engine, Mr. X finds a shopping store and visits the website. Upon reaching the website, he finds a popup ad about purchasing discount coupons that asks him to click to claim a discount. Now, when he clicks the advertisement, after certain redirects, he lands on a website that looks identical to a payment service website to pay using the card. He did not consider the website suspect due to the identical appearance of the website content and URL structure. If he enters his card details, the credentials will be stolen as it is a phishing website. Early evaluation of the website can save him from being a victim of the attack. If he uses our approach of early detection and classification as a browser extension, the efficient model will first evaluate the URL where the alluring ad redirects. If it contains anomalies, it will be flagged as a phishing link, and he will be notified. If the URL structure contains no anomalies, the next layer of text content evaluation will scrape the text content of the URL and classify the text based on natural language processing. The alluring spam text of the website will be detected by the text content layer and flagged as phishing. It is seen that there are websites that contain limited text content and use images instead of text. Suppose URL and text evaluation found no anomalies. In that case, the image evaluation layer will grab the images from the website. After text extraction from the images, it will be evaluated for classifying the website based on image text. If spam text exists, the model will label the website as suspected and it will be flagged as phishing. In this layered approach, comprehensive evaluation leaves minimum chances of a phishing website bypassing the detection model.

### 3.3. Datasets

The dataset for the phishing website URLs is obtained from the PhishTank website, which is a real-time updated forum of the reported phishing URLs. At PhishTank, the URLs of phishing websites are reported and again tested by others to label them as phishing ones. The PhishTank dataset containing 20,000 URLs is obtained and used. The URLs of legitimate websites are taken from Alexa by Amazon. The top 20,000 websites of daily use that are reported by Alexa are used as a reference for legitimate websites. As in the proposed research methodology, binary classification is considered because either the website will be for phishing or it will be legitimate. In this case, for the training of machine learning models, there is a requirement for the labeled data. PhishTank provides the phishing links labeled as malicious. The second class of binary classification in the proposed research is a legitimate website, and for that, labeled data from Alexa are obtained. Before the extraction of features, both these datasets are mixed up to avoid any biases that may affect the results.

In the second layer, the textual analysis of the website is required, and for that, the techniques of natural language processing are used. The extracted text from the website is tested in the model, which is trained using the online available dataset of SMS spam and ham dataset from Kaggle. As the spam or phishers use identical statements and text to lure the victim, the lexical and textual features of the SMS spam dataset are considered for training the model and testing the target website by using the text of that website.

### 3.4. URL Layer

There have been multiple types of research where machine learning models have been used for the classification of URLs and tagging them for being phishing URLs or legitimate URLs. In most such research, the authors considered the lexical features of the URLs. It is seen that the phishing URLs usually have certain characters like commas, slashes, question marks, hyphens, etc. Also, the suspected URLs are usually longer in length than the valid legitimate URLs. Previously, there were studies where only the lexical features were considered for the classification.

**Figure 3:**
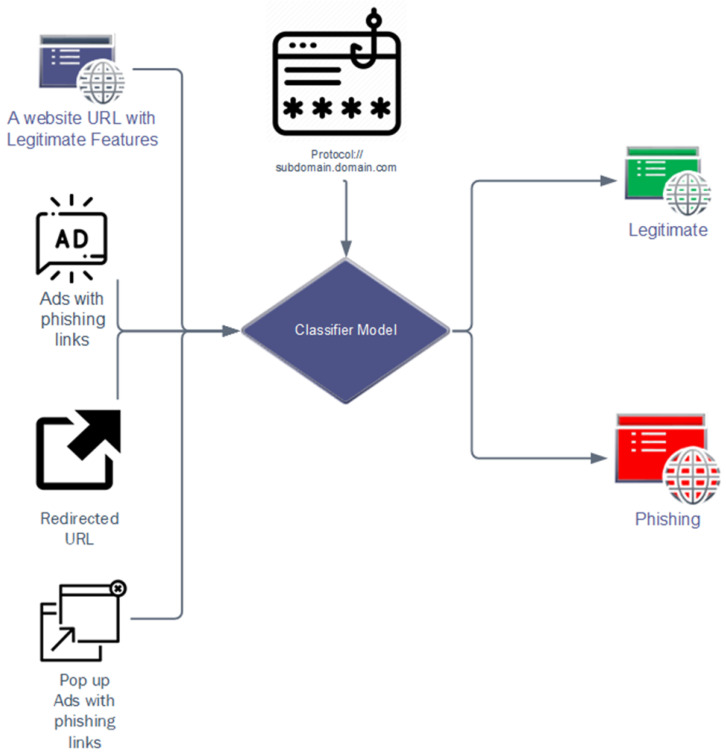
The classifier model predicts ham and spam according to URL features.

As shown in Figure 3, in the proposed methodology, not only the common lexical features of the URLs are considered but also the domain-based features of the URLs are focused to treat as features that are used to classify the valid and phishing URLs. The domain-based features include SSL security, the index number of URLs in the Google website index, domain creation time, redirects, URLs containing mailing addresses, etc.

Modern-day attackers are intelligently adapting their phishing websites to look more realistic. Modern phishing websites may have URLs just like valid websites without having alpha-numeric characters and they may also have valid domains and security. Spotting such websites has been a major challenge as the classifying models that are based on the lexical features fail to classify when the features of phishing URL are the same as the valid URLs. For such cases, there has been a requirement for some salient features. These salient features are possessed by legitimate websites and are usually not possessed by phishing websites. The quantity of redirects is the feature that is considered to be the salient feature as phishing websites are usually reported and taken down; hence, they have a shorter life span. For this reason, the attackers have to change the domain names to use the phishing links again or redirect to the old links through new links. To take the victim to a fake page, usually, the ads have to redirect the victim to some phishing links. To make this process look legitimate, the victim is usually redirected multiple times. This can be taken as the salient feature of collecting the number of redirects and if the number of redirects is more than certain normal numbers, the URL can be tagged as a phishing link.

In this proposed methodology, the previously used features as well as some new salient features are considered for the training of machine learning models. In total, the 22 salient features are considered for the classification model training, as shown in the Table 1.

### 3.5. Text Layer

In this layer, the text from the target website is scrapped and given as input to the machine learning model trained with the SMS spam and ham dataset obtained from Kaggle. The text is truncated, cleaned, and tokenized. This clean and ready-to-test text data are then introduced to the machine learning model. The model identifies the keywords, their previous word, and the coming word and analyzes the context to tell whether the website text lies in the spam context or the ham context. This text being given as input to the spam/ham-trained model described ensures the proper cleaning, tokenizing, and removing of stop words for better accuracy of the testing model. This acts as the second layer of the phishing detection layered approach. Some websites have legitimate URLs, but their body text contains texts and paragraphs to lure the victim to click certain phishing links. This text analysis layer of the proposed method will help the victim to stay safe from such attempts where the URL looks legitimate, but the body text of the website leads to a phishing attack. The dataset trains the model to classify the text input into the two classes of ham and spam. The suspiciously analyzed text is labeled as spam text, while the text showing normal and valid context is labeled as the ham text data. This can surely improve phishing detection as the use of natural language processing can analyze the context. Hence, the contextual analysis of the text can be proved as an extra layer of detection criteria for text-based phishing websites.

**Figure 4:**
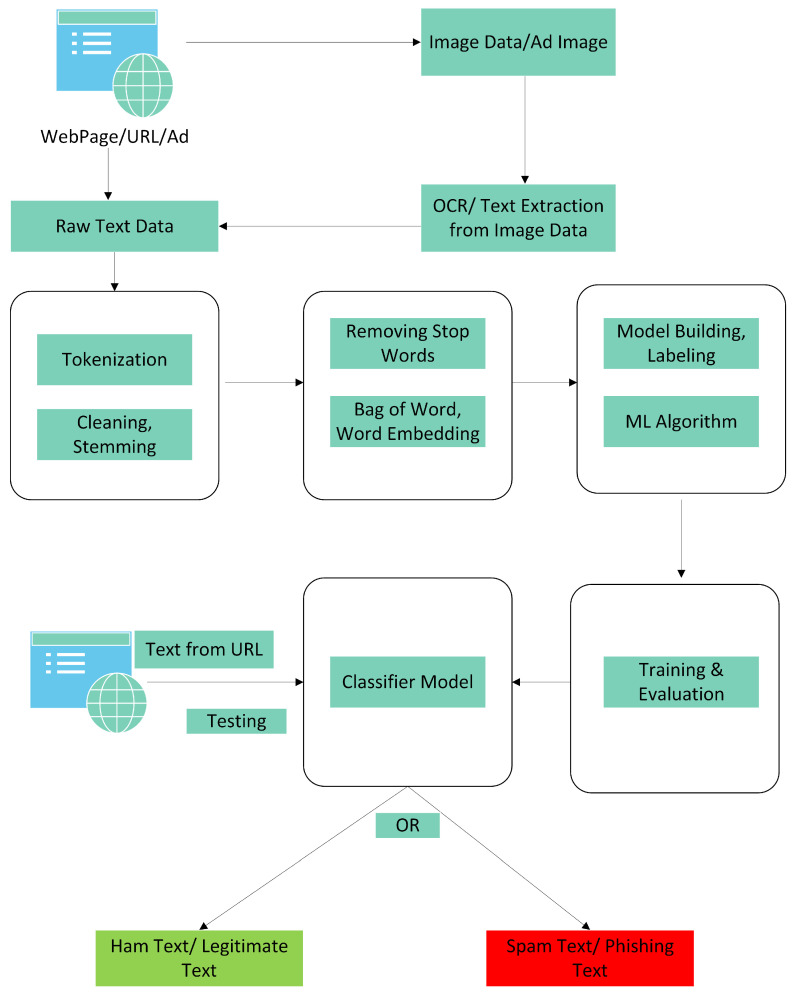
Figure shows processes of text scraping, pre-processing and classification.

Figure 4 elaborates on the text layer of the proposed layered model. In the first part of data collection, the website or advertisement containing the website URL is visited for scraping data. The text is collected from the website using a web scraping technique, while for the websites containing images, the target images are obtained, and text is extracted from them using the OCR approach. The collected text data are in raw form and require pre-processing. In the pre-processing phase, the text strings are tokenized, and text stemming is performed to keep a potentially useful set of words and remove any unnecessary and irrelevant data. Further, the stop words of the sentences of natural language are removed as they do not impact converging towards a class. In the training phase, the classification model is applied to the cleaned and labeled data for model training, and performance is evaluated. Once the training is completed, the classifier model is ready to test new URLs. The text data obtained from the website are provided to the classifier model as input to classify the website as a phishing or legitimate website.

### 3.6. Image Layer

The third layer of the proposed model is the layer in which the images from the website or advertisements are obtained and given as input to the image-to-text conversion module. It is seen that some phishing websites contain only one image or banner that contains text. Such websites do not have text data in their body as well, and they can usually have legitimate URLs. Such websites can be left undetected from the previously mentioned layers. Also, the focus is to detect phishing that lures the victim by showing alluring ads. It is seen that the ads that are popped up on the websites are in image formats having back-links that are opened upon clicking. The third layer of the proposed methodology will take these images as input to convert them into text so that they can be readable and given as input to the model presented in the second layer for the textual and contextual analysis of the images with the help of natural language processing. These images can be classified with the help of image processing techniques, but that will take a lot of processing as well as resources.

Converting the image into text is considered to be a useful and compact solution as it is evident that phishing ads and images usually have slang terms or talk about monetary benefits to lure the victim into clicking on them. For the execution of this layer, a Python library takes the image from the website and converts it into readable text. This text is then provided as input to the spam/ham-trained model described above in the text layer after the proper cleaning, tokenizing, and removing of stop words for better accuracy of the testing model. In this way, the model will treat the text to know the context, and then it will classify it as ham or spam data. The model is elaborated in Figure 4 above.

## 4. Performance Evaluation

The experimental setup included an Intel Core i7 processor (3.4 GHz, 8 cores) and 16 GB of RAM. The machine was running on the Windows 10 operating system and all the computations were performed using Python 3.9 in the Jupyter Notebook. The experimentation was performed in June 2023, utilizing the datasets of phishing and legitimate URLs taken from Alexa and PhishTank. In the initial phase, pre-processing of the datasets is performed. During pre-processing, all the entries are checked one by one to remove any empty entries in the dataset file. After this, 20,000 records are taken from both datasets and mixed randomly so that the biases can be avoided due to overfitting of the model. Also, In the next step, the proposed features are extracted using Python code executing in Jupyter Notebook. Each of the links is checked one by one automatically to extract the above-mentioned 22 features. The heat map of these features is also generated along with the histogram of each feature so that the statistical analysis can be performed before training the dataset in machine learning models. These features are then stored in a new file. After completion, the resultant file is again randomly mixed so that the phishing site feature entries and legitimate entries are mixed up to avoid any overfitting of the model. The pre-processed data are then trained to the machine learning algorithms by setting up data into classes of X_train, Y_train, X_test, and Y_test. The split data are then trained to the ML models.

In the same way, the ham and spam dataset is obtained from Kaggle, which is an online dataset provider. This dataset is again used to train the machine learning models to make it learn to classify any text input into spam or ham. The whole datasets were classified into binary values in the last column, which is denoted as the required resultant value. The cleaned and pre-processed datasets after randomized sorting were trained to the best machine learning classifier algorithms. The algorithms for the machine learning model were selected keeping in view that the proposed model can be an efficient and maximum accurate model for the classification and making predictions.

**Figure 5:**
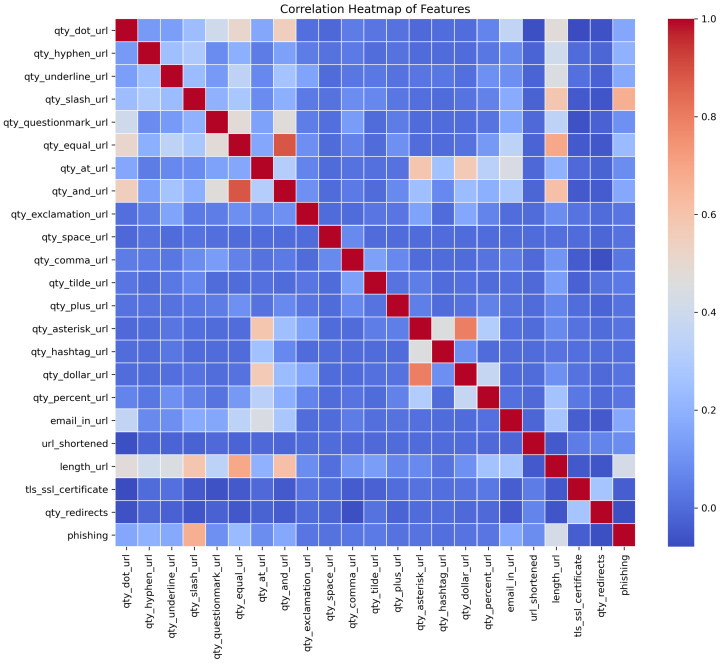
Heat map of the features is shown according to their binary values.

**Figure 6:**
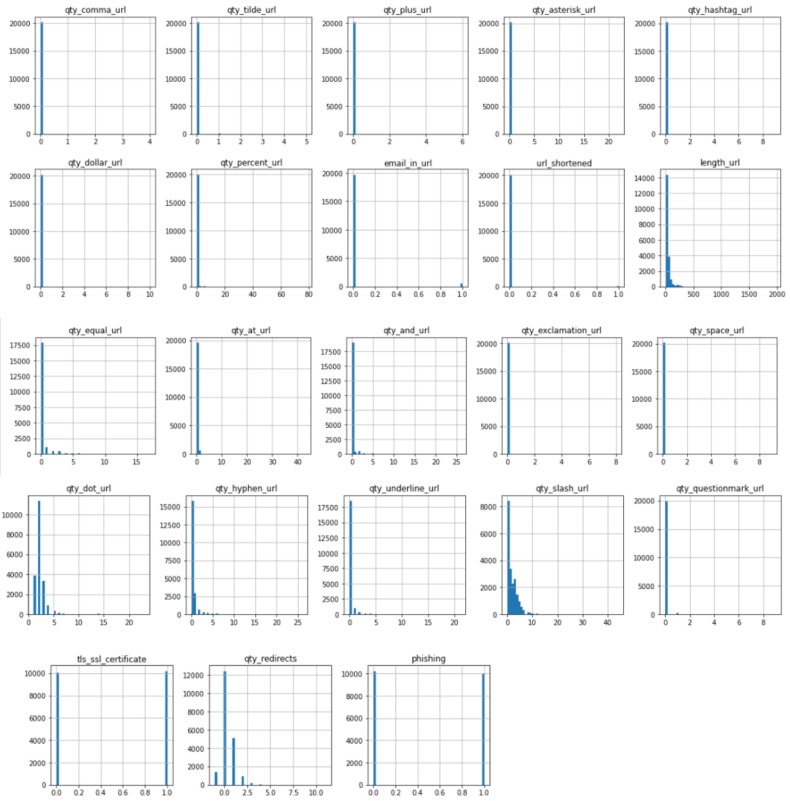
Histogram of features.

The process of cleaning the datasets and pre-processing for the feature extraction took much time as each of the links was required to check one by one for the feature extraction. As far as scalability is concerned, the machine learning models are trained on a large dataset to explore the performance on a large scale. The scalability of the solution is also evaluated on different websites that contain different lengths of URLs, sizes of text, and image content. Regarding the URL layer, the training data already contained websites with varying lengths of URL, and the feature extraction process raised no exceptions or delays for longer URLs as only the salient features are being evaluated in the target URL. The website text size may vary and it is also evaluated in terms of computational efforts and processing time. It is seen that the text content is usually in KBs. The model is designed to perform only the necessary steps of text pre-processing so that the feature extraction and classification can be optimized. This approach makes the solution practical as no major delays are seen in the testing and training phase during the text-based classification. As far as the scalability and performance of image content-based evaluation are concerned, the images are obtained in the least possible quality as text extraction from images works well even for less-quality images. This optimized the approach and took practical processing time for image extraction. Also, image text extraction and classification are prioritized as the third layer. For optimization, only those images are grabbed that have underlying hyperlinks because, in phishing websites, the underlying hyperlinks are there to lead the victim to the phishing web page. For scalability and optimization at a large scale, the model training is designed to label true or false bits for the selected features that help deal with the optimization of the trained model in terms of size. The statistical analysis of the features is performed after the pre-processing of datasets. The heat map and histograms are generated to check the impacts of different features according to their maximum, minimum, and frequency values in the final datasets. The histograms and heat map are shown in Figure 5 and Figure 6 below, respectively.

Figure 5 represents the statistical analysis of the dataset; we have developed a correlation heatmap of features. At the initial phase of the study, the correlation heatmap provides insight to estimate patterns and relationships of features to a specific class. The current research problem is a binary classification problem where the models must predict whether a URL belongs to a phishing or legitimate class based on features. For the estimation of patterns, the correlation heatmap helps check the impacts of each feature regarding its convergence towards a class. The intensity of colors denotes the value on a scale of 0.0 to 1.0. The dark red color at the diagonal is the maximum correlation value of 1 because each feature perfectly correlates with itself. The dark blue cells denote the minimum value of correlation 0, which means there is the least correlation and impact of that feature for converging toward a class. The darkness of the red color shows the strength of positive correlation, while the darkness of the blue color shows the strength of negative correlation according to correlation value. The light shades of dark and blue colors show a medium positive correlation and a medium negative correlation of a feature. The correlation heatmap of features is interpreted as the features with strong positive correlation tend to increase and may have characteristics of the same class. In contrast, the features with strong negative correlation mean they are opposite regarding their direction and inversely related to a class. In the same way, lighter shades represent comparatively weak associations with the target variable, but still, they have an impact. Analysis of the correlation initially provides an overview of feature importance, but further modeling and analysis are conducted to determine the final features.

In Figure 6, the histograms show the values of each feature according to the occurrence. This provides an overview of the value range for each feature. Once the features were extracted, the training phase did not take much time as the data were pre-processed according to the compatibility and least complexity of the algorithm application. For the application of the prediction model, the following algorithms were considered, and these are explained below.

### 4.1. Machine Learning Model Evaluation

The following machine learning models are applied on the prepared dataset.

#### 4.1.1. Decision Tree

The decision tree classifier algorithm is popular regarding its classification abilities. The important feature of capturing the descriptive data for the decision making from the given knowledge or data makes it an ideal classifier that divides nodes into sub-nodes like a tree, and the sub-nodes are divided in a way to ensure homogeneity of the results at the end [23,24]. In the proposed method, the features extracted from the datasets were provided as internal nodes, the branches of decision tree algorithms were supplied the rules of classification for the decision making, and the decision tree provided results according to the leaf nodes. The decision tree worked well and provided an accuracy of 91% in the case of training and an accuracy of 89.6% in the testing phase.

**Figure 7:**
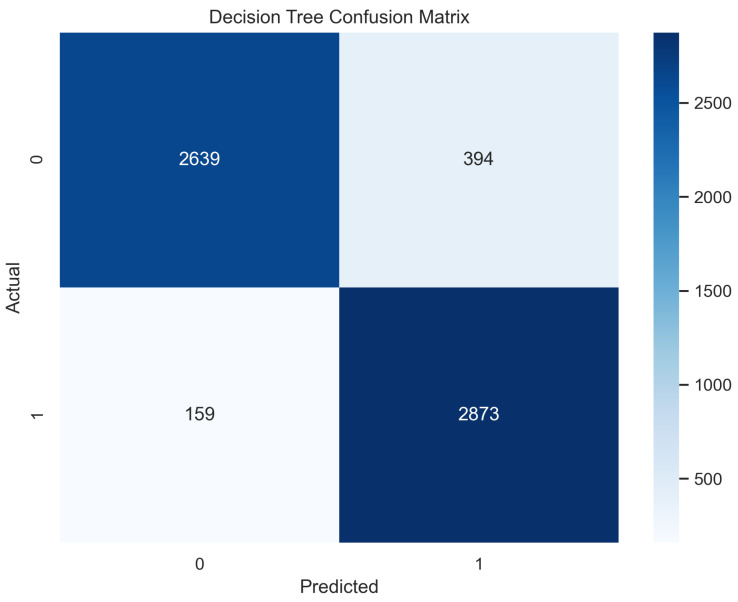
Confusion Matrix for Decision Tree.

**Figure 8:**
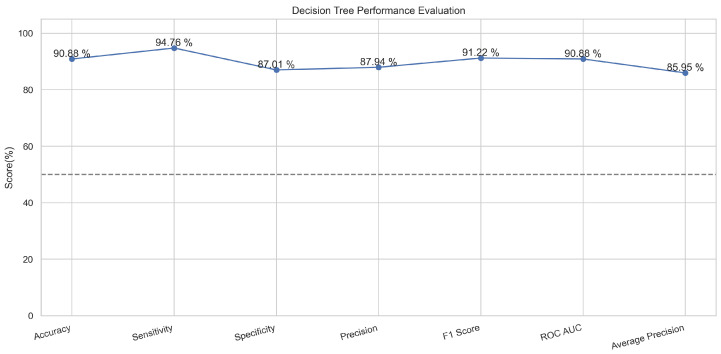
Performance Evaluation for Decision Tree.

In Figure 7, the true labels and predicted labels are shown in the matrix. The true positive, true negative, false positive, and false negative values are shown in the matrix cells for the test phase. In Figure 8, the performance evaluation for the decision tree model is provided. The high value of accuracy describes the model’s high level of overall correctness. With a higher sensitivity also known as recall, the model identified maximum positive instances and minimal skips. The high value of specificity exhibits that the model correctly identified the negatives. Similarly, the higher precision score presents the model’s performance in positive predictions. F1 score elaborates on the balance of precision and recall. The ROC AUC score exhibits the ability of the model to discriminate between classes. The average precision is provided to have an overview of the precision/recall trade-off.

#### 4.1.2. Random Forest

Random forest is another classifier used for binary classification and prediction according to the provided dataset. This algorithm includes multiple decision trees for different subsets of the provided dataset [25]. It takes an average value to improve the accuracy of the model for efficient prediction using each tree and on the basis of frequent results from these trees [26]. In the proposed model, the random forest learned the dataset and provided remarkable accuracy. It predicted 90.8% accurately in the training phase and 89.8% in the testing phase. In random forest, we tried multiple values to find the moderate value. According to our dataset, setting the maximum depth to a value of 5 provided good results. Setting a moderate value of maximum depth controls trees to attain generalization because, on a moderate value of maximum depth, each tree is relatively shallow and minimizes the chances of overfitting due to generalization.

**Figure 9:**
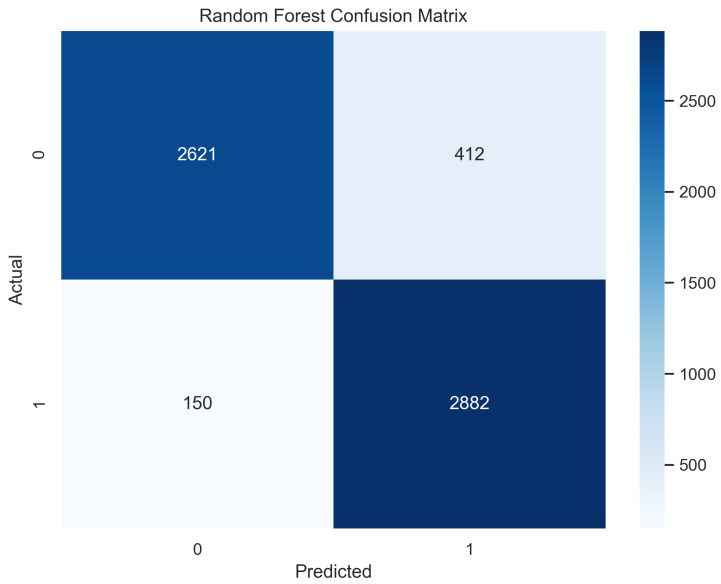
Confusion Matrix for Random Forest.

**Figure 10:**
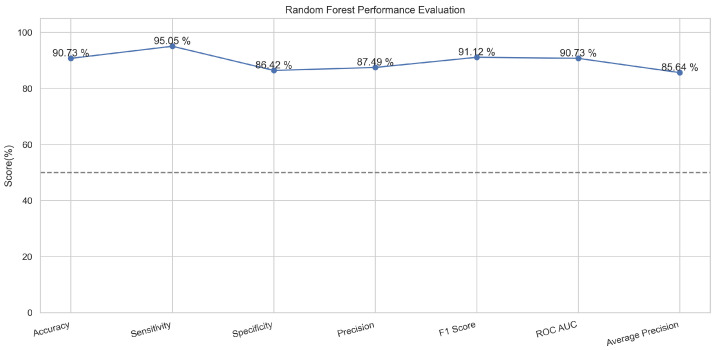
Performance Evaluation for Random Forest.

In Figure 9, the true labels and predicted labels are shown in the matrix for the random forest. The true positive, true negative, false positive, and false negative values are shown in the matrix cells for the test phase for the random forest classifier. In Figure 10, the performance evaluation for the random forest model is provided. The scores of accuracy, sensitivity, specificity, and precision provide an insight into the model’s overall correctness, ability to identify positive instances, the capability of correct identification of negatives, and performance in positive predictions, respectively. Similarly, the F1 score, ROC AUC, and average precision describe the balance of precision and recall, the ability of the model to discriminate between classes, and an overview of the precision/recall trade-off, respectively.

#### 4.1.3. Multilayer Perceptron

This neural network model uses a set of inputs to generate a set of outputs in a feed-forward way. This is a deep learning algorithm that uses back-propagation for remembering the knowledge from the training phase [27]. The various layers of the input nodes are connected among input and output layers in a directed manner [28]. This algorithm is used in the proposed method because it is a supervised learning algorithm based on neural networks in the category of deep learning. The multilayer perceptron worked well and predicted the results with an accuracy of 91.8 in the training phase and an accuracy of 90.9% in the case of testing. In MLP, we kept a random seed to a specific value of 0 to attain consistent results on each run during the training and testing phases. The small L2 regularization, parameterized as alpha in the MLP algorithm, is set to a minimum so that the model can be generalized and overfitting can be avoided. The initial learning rate we provided to the model is 0.001. The maximum iteration value is kept at 500 so the model can converge gradually but up to a reasonable limit.

**Figure 11:**
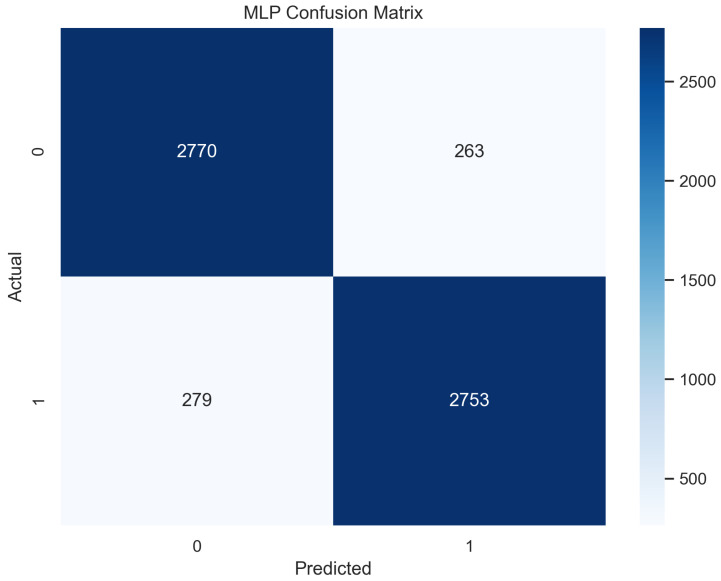
Confusion Matrix for MLP.

**Figure 12:**
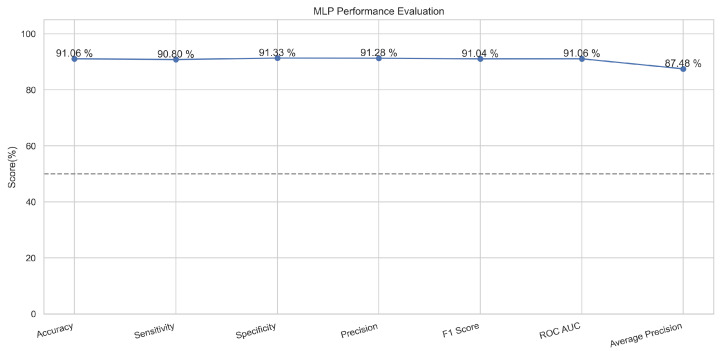
Performance Evaluation for MLP.

In Figure 11, the true labels and predicted labels are shown in the matrix for the MLP algorithm. The true positive, true negative, false positive, and false negative values are shown in the matrix cells for the test phase. In Figure 12, the performance evaluation for the MLP is provided. The scores of accuracy, sensitivity, specificity, and precision provide an insight into the model’s overall correctness, ability to identify positive instances, the capability of correct identification of negatives, and performance in positive predictions, respectively. Similarly, the F1 score, ROC AUC, and average precision describe the balance of precision and recall, the ability of the model to discriminate between classes, and an overview of the precision/recall trade-off, respectively.

#### 4.1.4. Support Vector Machine

The support vector machine algorithm is used for linear or non-linear problems in the supervised machine learning classification or regression usually. This algorithm works by drawing a line or hyperplane. This line and hyperplane divide the data into binary classes [29]. The points with major or distinct differences are aligned away from the line on both sides, while the points having little feature difference are placed closer to the line on both sides [30]. This is a popular machine learning classifier that performed well in our proposed method. It provided an accuracy of 88.9% in the case of the training and 87.8% in the testing phase.

**Figure 13:**
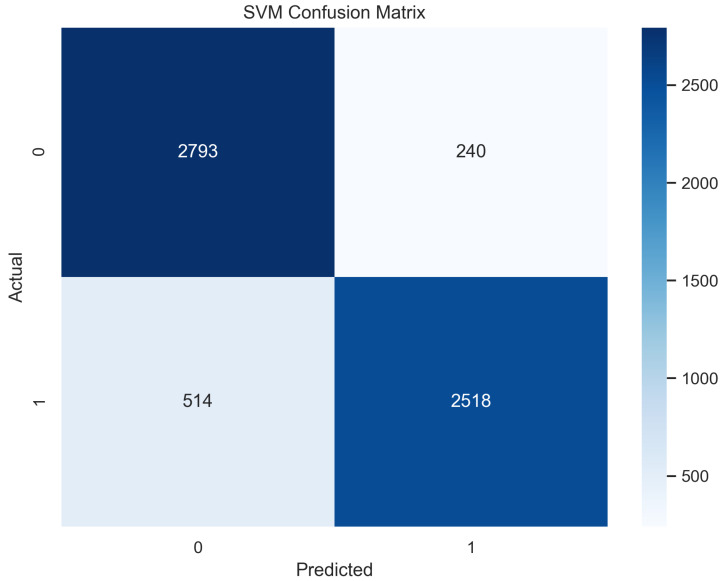
Confusion Matrix for SVM.

**Figure 14:**
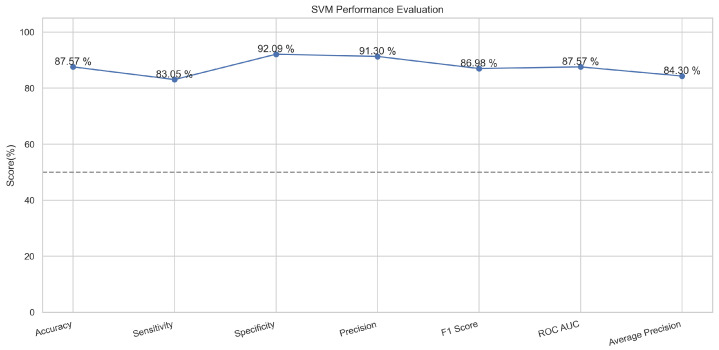
Performance Evaluation for SVM.

In Figure 13, the true labels and predicted labels are shown in the matrix for the SVM algorithm. The true positive, true negative, false positive, and false negative values are shown in the matrix cells for the test phase. In Figure 14, the performance evaluation for the SVM is provided. The scores of accuracy, sensitivity, specificity, and precision provide an insight into the model’s overall correctness, ability to identify positive instances, the capability of correct identification of negatives, and performance in positive predictions, respectively. Similarly, the F1 score, ROC AUC, and average precision describe the balance of precision and recall, the ability of the model to discriminate between classes, and an overview of the precision/recall trade-off, respectively.

#### 4.1.5. Logistic Regression

A logistic function is implemented in this algorithm, which provides only two values based on the features [31]. These values are usually in the form of true or false. This is a common classifier that classifies data by implementing the logistic function [32]. In our experimentation, the logistic regression model performed well and predicted up to 87.5% in training mode and 86.5% in the testing mode.

**Figure 15:**
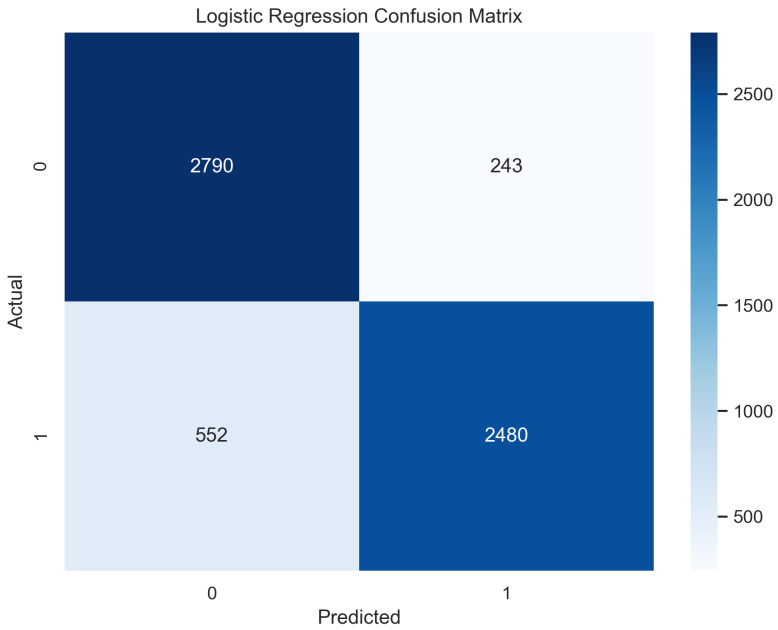
Confusion Matrix for Logistic Regression.

**Figure 16:**
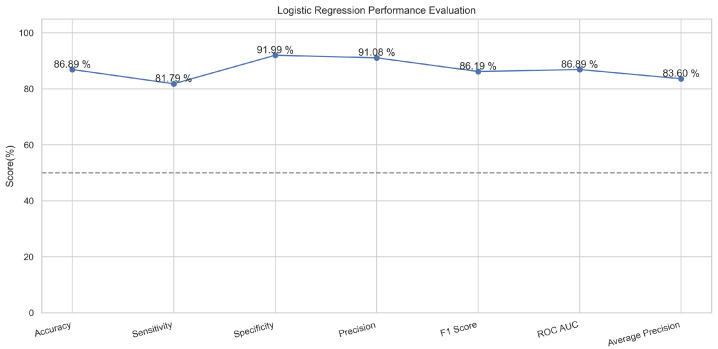
Performance Evaluation for Logistic Regression.

In Figure 15, the true labels and predicted labels are shown in the matrix for the LR algorithm. The true positive, true negative, false positive, and false negative values are shown in the matrix cells for the test phase. In Figure 16, the performance evaluation for the random forest model is provided. The scores of accuracy, sensitivity, specificity, and precision provide an insight into the model’s overall correctness, ability to identify positive instances, the capability of correct identification of negatives, and performance in positive predictions, respectively. Similarly, the F1 score, ROC AUC, and average precision describe the balance of precision and recall, the ability of the model to discriminate between classes, and an overview of the precision/recall trade-off, respectively.

#### 4.1.6. XG Boost

Extreme gradient boosting (XGBoost) is a classifier that works on the decision tree approach. The tree has nodes and sub-nodes is boosted downwards to divide further according to the features [33]. The XGBoost model outperformed all other algorithms applied due to its compatibility and best fitting for the tabular datasets [34]. The performance evaluation describes that for the structured data in the supervised machine learning, this algorithm model worked best as compared to others, with an accuracy of 94.1% in the training phase and 91.2% in the testing mode. In the XGBoost model, we passed parameter values as follows for optimum run and good accuracy according to our dataset: For optimized execution time and handling overshooting, we tried multiple values for learning rate, and we found the best performance when the value was set to 0.4. The said learning rate value also ensured faster convergence and provided optimum results. The slightly greater value of 7 for maximum depth handled complexities in data more efficiently. Also, we set label encoding to false so that the variables of more than one category can be handled. Considering the nature of the problem, we selected logarithmic loss to make it relevant to the classification problem.

**Figure 17:**
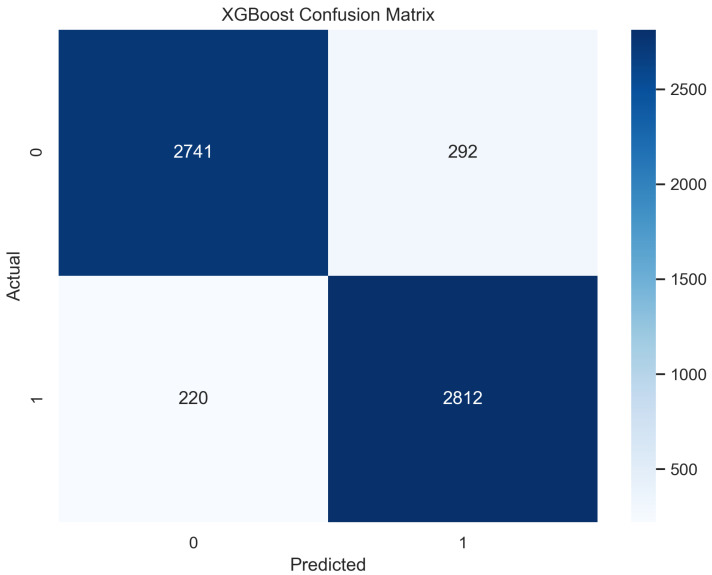
Confusion Matrix for XGBoost.

**Figure 18:**
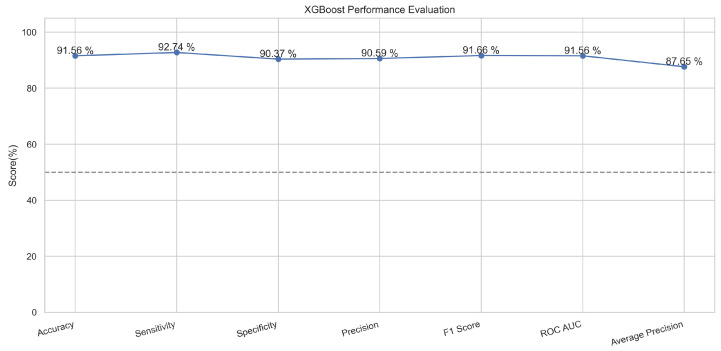
Performance Evaluation for XGBoost.

In Figure 17, the true labels and predicted labels are shown in the matrix for XGBoost algorithm. The true positive, true negative, false positive and false negative values are shown in the matrix cells for the test phase. In Figure 18, the performance evaluation for the XGBoost model is provided. The scores of accuracy, sensitivity, specificity, and precision provide an insight into the model’s overall correctness, ability to identify positive instances, the capability of correct identification of negatives, and performance in positive predictions, respectively. Similarly, the F1 score, ROC AUC, and average precision describe the balance of precision and recall, the ability of the model to discriminate between classes, and an overview of the precision/recall trade-off, respectively.

### 4.2. Text Analysis with NLP Techniques

For the text analysis according to the natural language processing techniques, the web text was taken from the target website and inputted in the testing model, which contained training knowledge from the ham and spam dataset with binary classes of ham and spam. Before giving the web text as input, it was cleaned and tokenized, and the stop words were removed for enhanced accuracy. The following algorithm models were applied for the text classification.

#### 4.2.1. Naïve Bayes

This algorithm works on a basic probabilistic approach as it makes predictions by analyzing the probability of an object learned from the given knowledge. In the natural language, the frequency of words occurring one after another is considered for training to this model so that it can learn the probability of different words coming before and after a word [35]. With the help of this feature, the algorithm learned probability. According to the classified dataset training knowledge, the algorithm learned to classify the combination of words to predict for belonging to spam class or ham class. In our contextual analysis model, the naïve Bayes provided a text accuracy of 91.19%.

**Figure 19:**
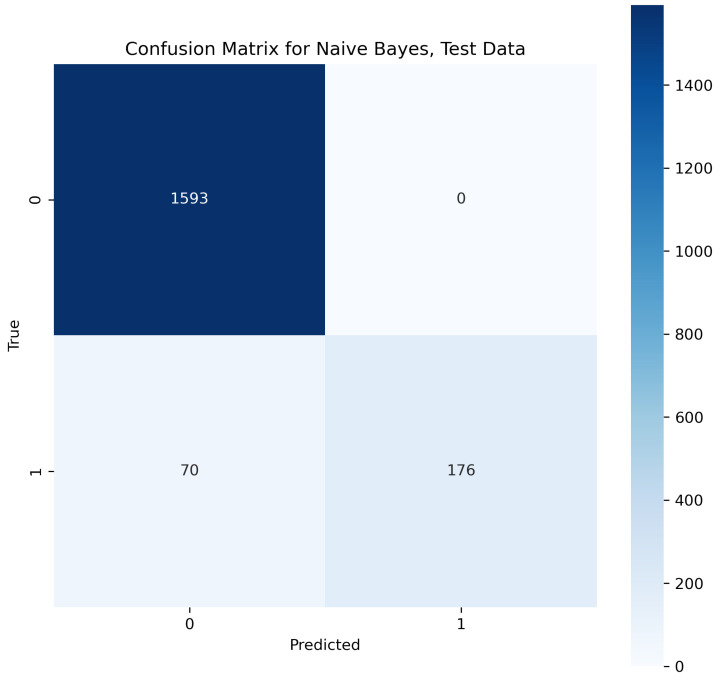
Confusion Matrix for Naive Bayes.

In Figure 19, the true labels and predicted labels are shown in the matrix for the naïve Bayes algorithm. The true positive, true negative, false positive and false negative values are shown in the matrix cells for the test phase.

#### 4.2.2. Linear SVC

This algorithm is the extended form of SVM explained above. The linear support vector classifier (Linear SVC) algorithm takes the data to be fitted upon, and then it returns a best-fitting scale or parameters according to the features trained. Once the model is trained and the hyperplane is according to the features, the algorithm starts fitting the coming input if it has relevant features upon which the hyperplane is based [36]. This makes it a popular and best classifier. In the text analysis of the second layer in our proposed methodology, the performance evaluation indicates that SVC outperformed and provided an accuracy of 98.9% for the testing phase.

**Figure 20:**
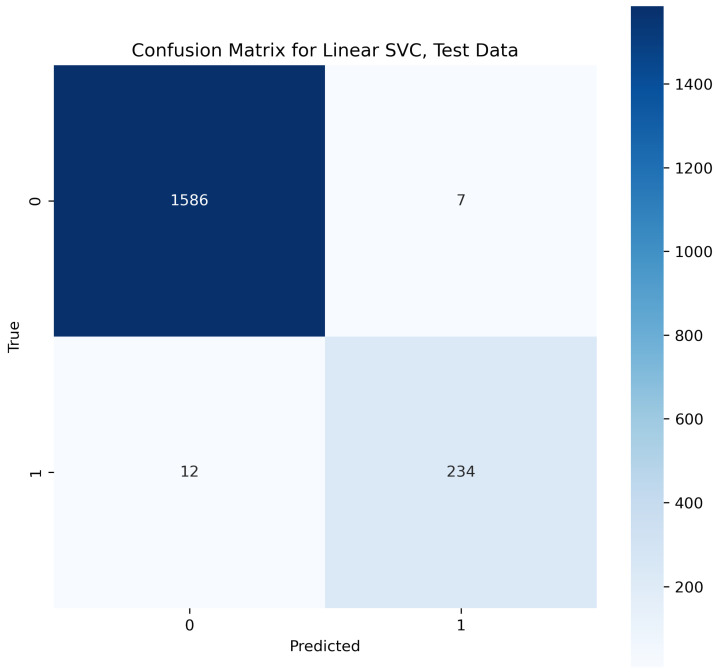
Confusion Matrix for Linear SVC.

In Figure 20, the true labels and predicted labels are shown in the matrix for the Linear SVC algorithm. The true positive, true negative, false positive, and false negative values are shown in the matrix cells for the test phase.

### 4.3. Performance Comparison

**Figure 21:**
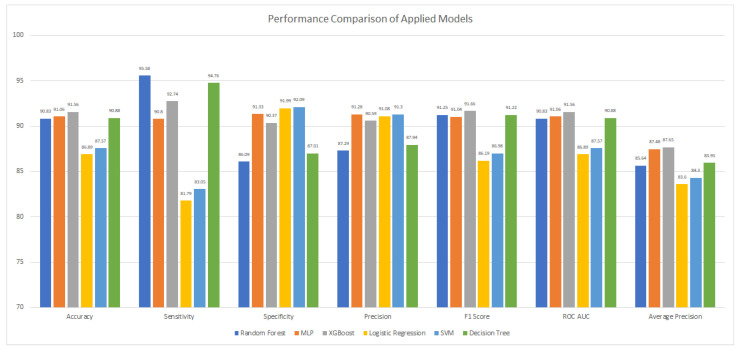
Performance Comparisons of Applied Models.

An overall comparison of the training and testing accuracy, precision, recall, and F1 score of the applied algorithms for URL classification is provided in Table 2 and Figure 21, which show that the XGBoost algorithm remained best in the training as well as a predictive model as compared to the other applied predictive classifiers.

The overall comparison of text classification algorithms is provided in the table and figures below.

**Figure 22:**
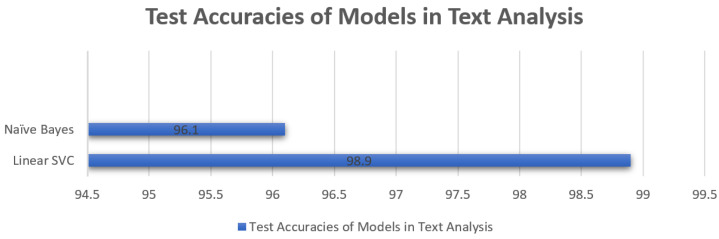
Predictive Model Accuracy Comparison Graph for Text-Based Analysis.

The comparison of accuracy is provided in Table 3 and Figure 22 for the accuracy of naïve Bayes and Linear SVC for the testing phase shows that Linear SVC outperformed with an accuracy of 98.9%. In the text analysis of the second layer in our proposed methodology, the performance evaluation indicates that SVC outperformed Linear SVC. In the third layer, the Python script efficiently takes an image from the targeted website and converts it into text. This text was again tokenized and cleaned by removing stop words and inputted in the text analysis model for the classification of the test according to the ham and spam model and similar accuracies of predictions were seen as described above for text analysis.

We tried to address the chances of uncertainties by ensuring optimized and proper feature engineering. Only the salient features are selected in the feature extraction phase, and these features are selected after sufficient research about the characteristics of phishing websites that differ from those of legitimate websites. These differences are considered anomalies after the detailed analysis of the characteristics of both classes. Further, the feature importance of each algorithm is considered to analyze the working of algorithms by checking on which basis the algorithm labeled a website as phishing. This helped us understand which aspects of our model evaluate the target website. Also, there were chances of data imbalance as the legitimate websites are much larger in number than the phishing websites. To handle this issue, we have shuffled multiple times to mix up the phishing and legitimate websites to avoid data imbalance and biasing.

### 4.4. Performance Optimization and Scalability

The machine learning models are trained on a large-size dataset to explore the performance on a large scale. The solution’s scalability is evaluated on websites containing different URL lengths, text sizes, and image content. Regarding the URL layer, the training data already contained websites with varying lengths of URLs, and the feature extraction process raised no exceptions or delays for longer URLs as only the salient features are being evaluated in the target URL. The website text size may vary and is evaluated regarding computational efforts and processing time. It is seen that the text content is usually in small size. The model is designed to perform only necessary text pre-processing steps to optimize the feature extraction and classification. This approach makes the solution practical as no major delays are seen in the testing and training phase during the text-based classification.

Regarding the scalability and performance of image content-based evaluation, the images are obtained in the least possible quality as text extraction from images worked well even for lower-quality images. This optimized the approach and took practical processing time for image extraction. Also, image text extraction and classification are prioritized as the third layer. For optimization, only those images with underlying hyperlinks are grabbed because, in phishing websites, the underlying hyperlinks are there to lead the victim to a phishing webpage. For scalability and optimization at a large scale, the model training is designed to label true or false bits for the selected features that help optimize the trained model in terms of size.

## 5. Conclusions

A multilayered evaluation approach based on machine learning was recommended and experimented with that enabled the end user to classify the advertisements and websites into legitimate and phishing websites for phishing detection. Different machine learning as well as deep learning algorithms were trained with the datasets according to the salient features. The predictive models performed well in predicting the websites as phishing or legitimate on the basis of their URL features in first layer of evaluation. In this layered approach, the second layer was about the text analysis of the advertisement or website. The available dataset is trained for the prediction of the text through contextual analysis using the natural language approaches. The predictive models successfully classified the text into ham or spam according to the trained features. In the third layer, the advertisement and website images were converted into text using the Python library of image-to-text conversion. The extracted text is provided to the second layer of the proposed model. The model again successfully classified the text into spam and ham according to the contextual analysis of the natural language processing approach. Overall, our research introduced an effective machine-learning-based approach for detecting modern phishing attacks by evaluating URL features, text content, and image content. Our model, particularly XGBoost, has shown promising results with high accuracy and precision.

However, it is essential to consider certain areas for further considerations. The evolving nature of phishing attacks requires continuous adaptation of our detection approach. The applicability of our approach in the real world can be more optimized by additions of handling zero-day attacks. Also, we have optimized the model regarding computational efficiency, but still there is opportunity for further optimization of image-to-text conversion for making the detection process robust. Future directions for research should involve adversarial testing to assess model vulnerabilities so that real-time mitigation can be made possible. Also, enhancing the user education regarding phishing attacks can also help avoiding such attacks. In conclusion, our research provided an optimized and efficient foundation for phishing detection using layered evaluation of a website.

## Figures and Tables

**Table 1:** Salient URL Features Selected.

No.	Feature	Description
1	qty_dot_url	Number of dots (.)
2	qty_hyphen_url	Number of hyphens (-)
3	qty_underline_url	Number of underlines (_)
4	qty_slash_url	Number of slashes (/)
5	qty_questionmark_url	Number of question marks (?)
6	qty_equal_url	Number of equality symbols (=)
7	qty_at_url	Number of at symbols (@)
8	qty_and_url	Number of ampersand symbols (&)
9	qty_exclamation_url	Number of exclamation symbols (!)
10	qty_space_url	Number of spaces ( )
11	qty_comma_url	Number of commas (,)
12	qty_tilde_url	Number of tilde symbols (~)
13	qty_plus_url	Number of plus symbols (+)
14	qty_asterisk_url	Number of asterisk symbols (*)
15	qty_hashtag_url	Number of hashtag symbols (#)
16	qty_dollar_url	Number of dollar symbols ($)
17	qty_percent_url	Number of percent symbols (%)
18	email_in_url	Email address in URL
19	url_shortened	URL shortening service used or not
20	length_url	Number of characters of URL
21	tls_ssl_certificate	HTTP or HTTPS
22	qty_redirects	Number of redirects

**Table 2:** Predictive Model Accuracy Comparison.

Sr No.	ML Model	Train Accuracy	Test Accuracy
1	Decision Tree	0.910	0.904
2	Random Forest	0.907	0.906
3	Multilayer Preceptrons	0.916	0.912
4	XGBoost	0.940	0.912
5	Logistic Regression	0.873	0.871
6	SVM	0.885	0.885

**Table 3:** Accuracy Comparison for Text Analysis.

No.	Algorithm	Accuracy
1	Linear SVC	98.9%
2	Naïve Bayes	91.19%

## Abbreviations

The following abbreviations are used in this manuscript:

SVM	Support Vector Machine
SVC	Support Vector Classifier
MLP	Multilayer Perceptron
ML	Machine Learning
XGBoost	Extreme Gradient Boosting
LR	Logistic Regression
DT	Decision Tree
RF	Random Forest
